# Supplementary material for: Diflubenzuron Induces Cardiotoxicity in Zebrafish Embryos
Source: Int J Mol Sci. 2022 Oct 8;23(19):11932. doi: 10.3390/ijms231911932 (PMC9570284; doi:10.3390/ijms231911932)
Supplement: Supplementary file 1 [file ijms-23-11932-s001.zip › ijms-1914659-supplementary.pdf]

Figure S1

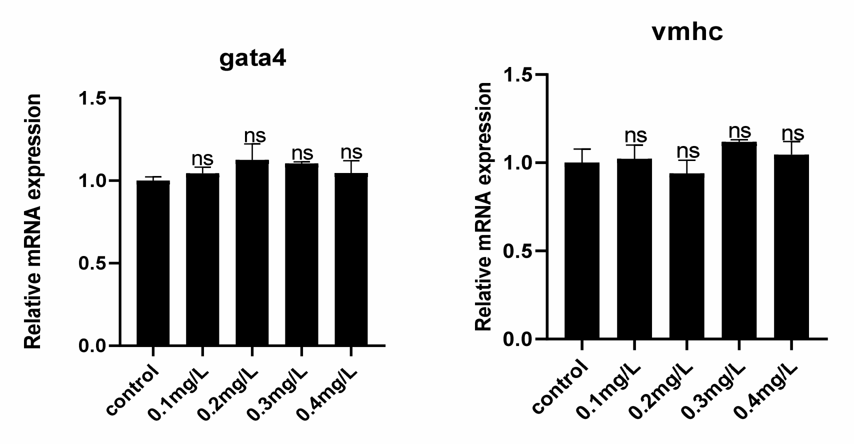

Figure S1 Effect of diflubenzuron exposure on mRNA expression of the genes related to heart development

The transcription of *gata4* and *vmhc* in zebrafish embryos after exposure to various concentrations of diflubenzuron (0, 0.1, 0.2,0.3 and 0.4 mg/L) at 96 hpf. Each bar represents mean ± SD (n=24) of three independent experiments. *ns: no significant difference.*

Figure S2

(A)

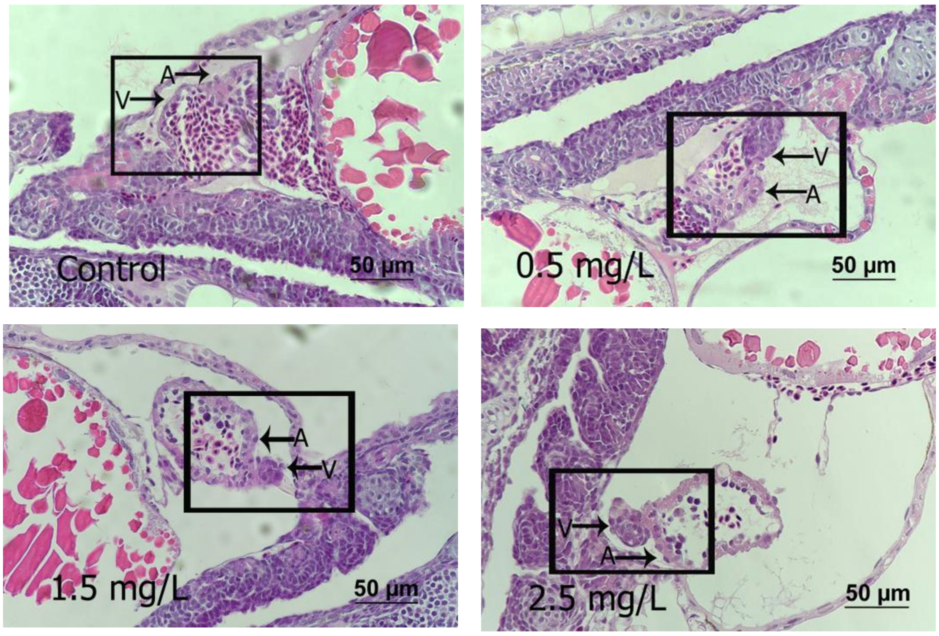

(B)

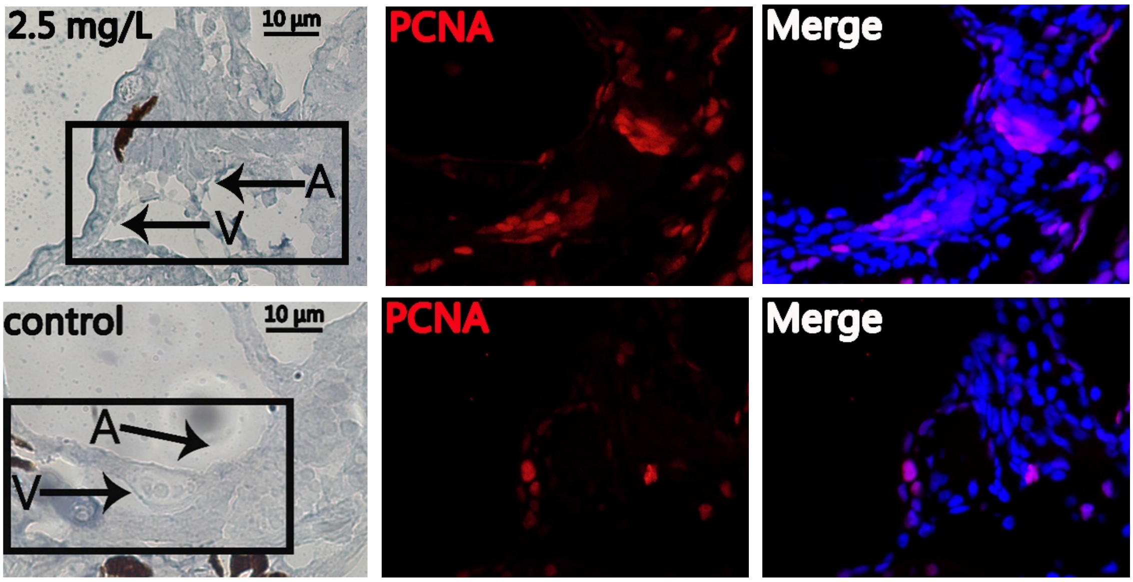

Figure S2 Exposure to diflubenzuron induces cardiac developmental toxicity in zebrafish embryos.

H&E staining of heart sections in control and 0.5, 1.5 and 2.5 mg/L diflubenzuron-exposed zebrafish at 96 hpf **(A)**, The heart slices were prepared, incubated with PCNA

primary antibodies and stained with cy3-tagged second antibodies **(B)**. DAPI was used to stain the nucleus. Each of experiment was repeated at least three times.

Figure S3

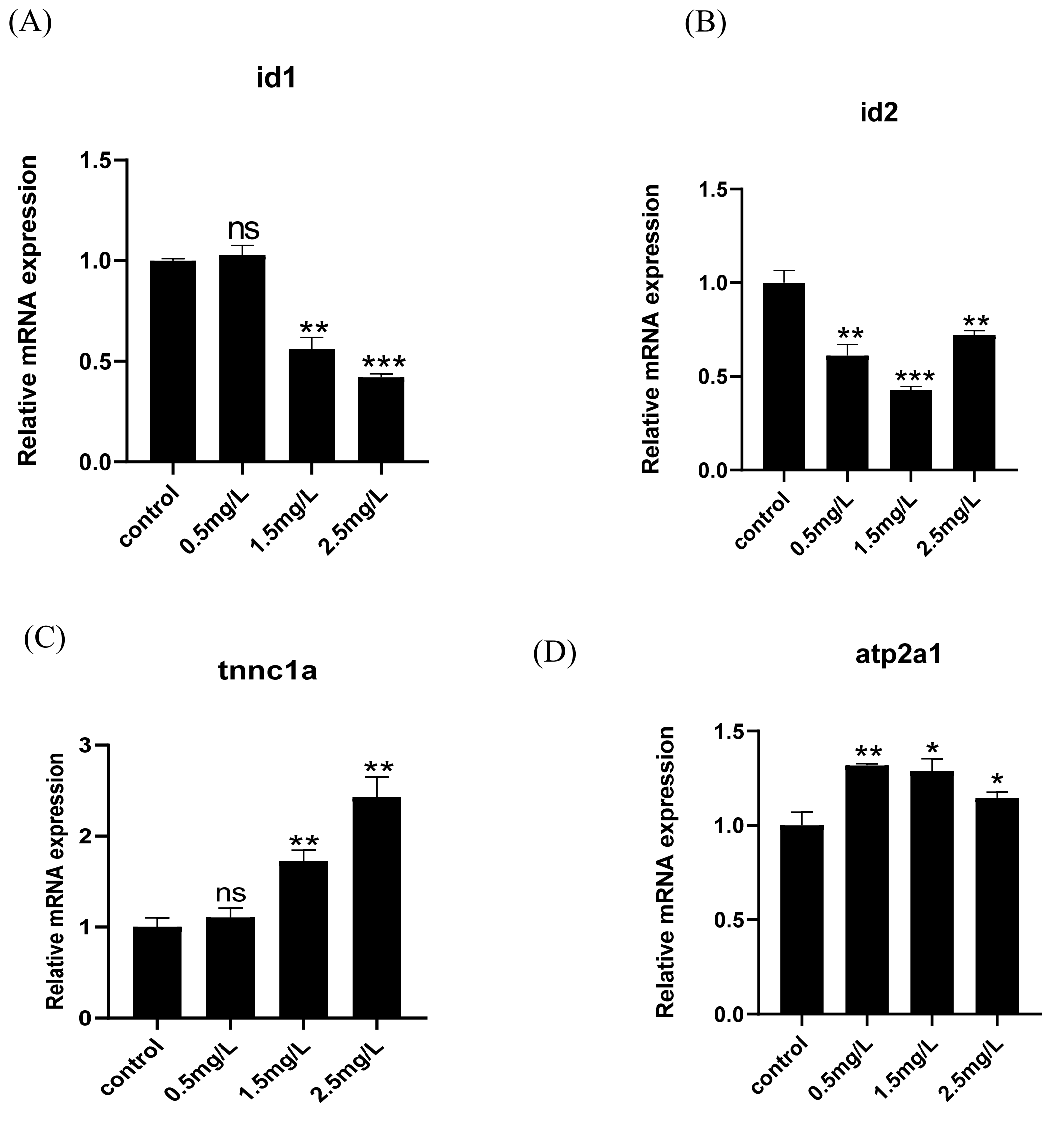

Figure S3 Exposure of diflubenzuron affects the gene expression of embryos in BMP signaling pathway and calcium dependent signaling pathway

Q-PCR was used to detect the genes in BMP signaling pathway (id1 and id2) **(A and B)** and calcium dependent signaling pathway (tnnc1a and atp2a1) **(C and D)**. Each bar represents mean ± SD (n=24) of three independent experiments. Asterisks indicate significant differences from control, *$p < 0.05$, **$p < 0.01$, ***$p < 0.001$.*

Table S1

Sequences of the qRT-PCR primers used in this study.

Target gene	Prime sequence (5'-3')
β-actin-F	AGCACGGTATTGTGACTAACTG
β-actin-R	TCGAACATGATCTGTGTCATC
nppa-F	GCTCCTGGTTTGGCAGCAG
nppa-R	GAGCTGCTGCTTCCTCTCGG
gata4-F	CTACAGGCACCCCAGCAGA
gata4-R	AGAGCCCGAGACCCGAAAT
vmhc-F	GAGCTTGATGAGGCAGAAG
vmhc-R	CAGCATAACGGAGACACAG
tbx5-F	AGTGACAGCGAACCAAAAG
tbx5-R	GTCTGGATGGACATAAAGC
myh6-F	CACCAGCAGACACTGGATG
myh6-R	GCTCCAAGTCCATTCTGAC
tbx2b-F	GGGGAACAATGGATGGCTAA
tbx2b-R	CCTAAGTGGGCTGGAAACC
klf2a-F	CCGTCTATTTCCACATTTTCG
klf2a-R	TCCAGTTCATCCTTCCACCT
bax-F	TCGAACATGATCTGTGTCATC
bax-R	TATGGCTGGGGTCACTTTTCTC
bcl2-F	TGGCGTCCCAGGTAGATAAT
bcl2-R	ACCGTACATCTCCACGAAGG
p53-F	CCCGGATGGAGATAACTTG
p53-R	CACAGTTGTCCATTCAGCAC
casepase3-F	GAGACCGCTGCCCATCACTAG
casepase3-R	ATCCTTTCACGACCATCT
casepase9-F	GCCTTTCTTGATTCCCTGCG
casepase9-R	TCTTGGCCTGGTTGGTCTCA
id1-F	CAGCAAAGTTGGAGGAGAGG
id1-R	AGCCGTTCTCCACAGAGATGCT
id2-F	GAGTGTAACGACGACGGAGC
id2-R-F	TGATGCAGGCTGGTTATCGC
bmp4-F	GGTCATTTTATTATGCCAAGT
bmp4-R	AAACGAATCGCAGAGGAGT
atp2a1-F	CCGACAAAACTGGCACCTTG
atp2a1-R	CTGCAGTCAACTTTGGCACC
tnnc1a-F	GGCAGAGCAACTCACCGAT
tnnc1a-R	GTAGGGTTCTGGCCCAACAT
